# AttenUNeT X with iterative feedback mechanisms for robust deep learning skin lesion segmentation

**DOI:** 10.1038/s41598-025-23830-1

**Published:** 2025-11-19

**Authors:** E. Babu, S. Murali

**Affiliations:** School of Computer Science and Engineering, VIT University, Vellore, Tamil Nadu India

**Keywords:** Deep learning, Attention UNet-X, Skin lesion, Segmentation, Feedback loop, Cancer imaging, Image processing, Skin cancer, Vitiligo

## Abstract

Accurate skin lesion segmentation is critical for improving early diagnosis of skin cancer. In this study, we propose AttenUNeT X, a novel extension of the U-Net architecture that integrates three key enhancements: (i) a feedback mechanism within decoder blocks to iteratively refine spatial features, (ii) a custom Order Statistics Layer (OSL) to capture extreme-value lesion patterns, and (iii) enhanced attention modules to prioritize diagnostically relevant regions. These progresses improve segmentation performance by allowing for a targeted reaction to important lesion features. These additions work in synergy to improve boundary precision and contextual learning. The model was trained and validated using the International Skin Imaging Collaboration (ISIC 2018) Dataset, with PH2-Pedro Hispano Hospital Dataset was experimented for comparative analysis and ISIC 2017 used for external testing and cross-validation, respectively and guarantee dependable performance across a range of images distributions. Our preprocessing pipeline included hair removal, resizing, normalization, and extensive data augmentation to promote robustness. To facilitate model generalizability, the preprocessing pipeline featured data augmentation and images enhancement, and the attention-augmented encoder-decoder layers of the design highlighted key lesion features. Experimental results demonstrate strong performance, achieving a Dice coefficient of 0.9211, Intersection over Union (IoU) of 0.8533, and pixel accuracy of 0.9824 on ISIC 2018. Similarly high metrics were observed on PH2 and ISIC 2017 Datasets. These outcomes validate the proposed model’s potential for reliable deployment in clinical dermatological workflows.

## Introduction

Skin cancer, particularly melanoma, remains a critical public health challenge due to its rising incidence and mortality. Early and accurate detection substantially improves treatment outcomes, yet dermoscopy-based diagnosis is often limited by subjectivity and variability among clinicians. To address these challenges, computer-aided diagnosis (CAD) systems leveraging deep learning have become increasingly important for reliable and automated skin lesion analysis.

**Fig. 1:**
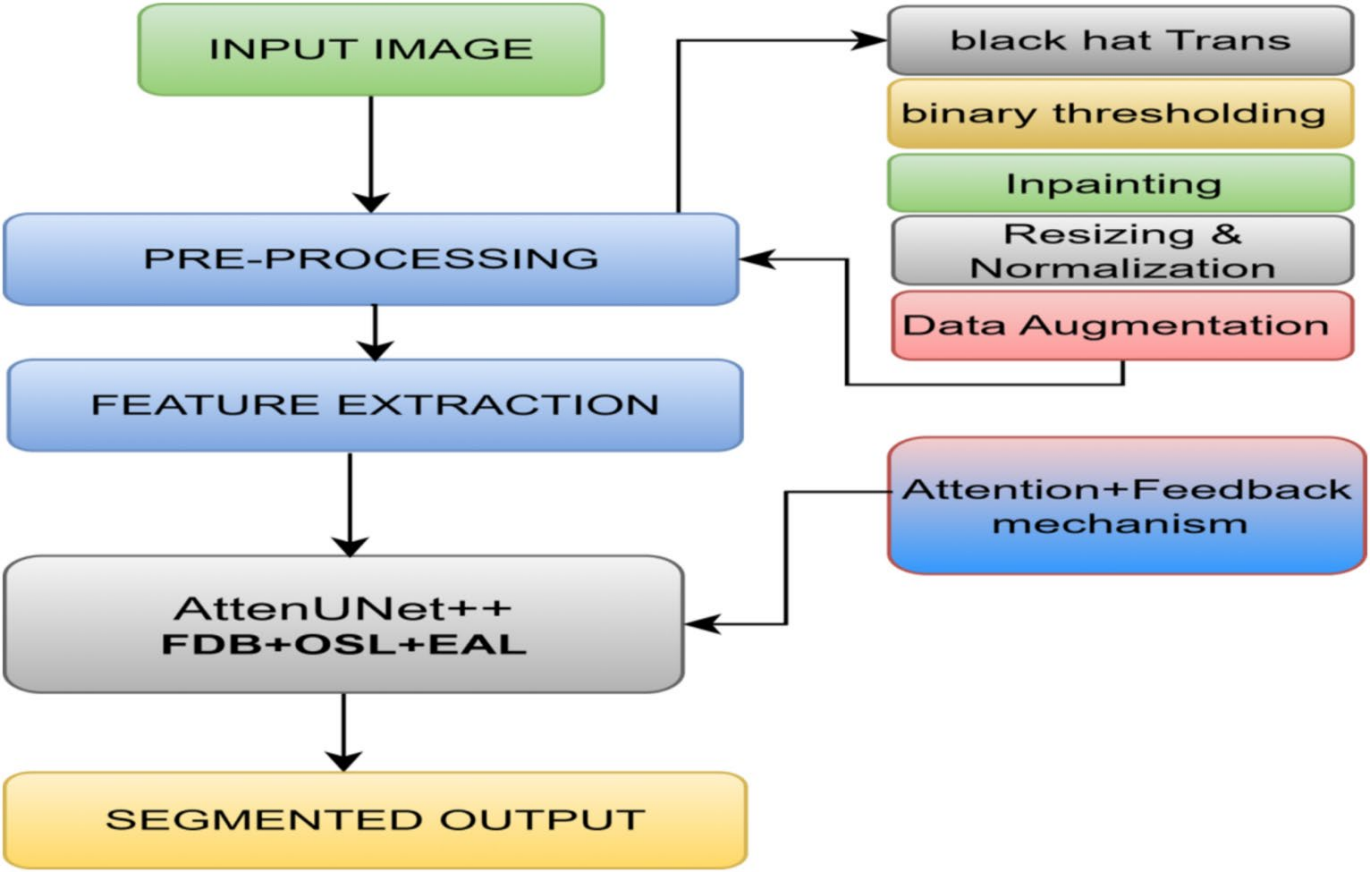
Overview of the proposed AttenUNeT X framework showing the preprocessing, encoder–decoder pipeline, and iterative feedback mechanism.

Among segmentation frameworks, U-Net and its variants dominate biomedical imaging owing to their encoder–decoder structure and skip connections. Enhancements such as attention mechanisms and multi-scale feature fusion have further improved boundary detection and texture representation. However, existing segmentation models continue to face three major challenges: (i) limited ability to refine lesion boundaries during decoding, (ii) poor capture of extreme features such as irregular color and shape outliers, and (iii) insufficient contextual attention to subtle lesion regions. To overcome these limitations, we propose AttenUNeT X, a refined U-Net architecture that introduces three complementary modules: (i) an iterative feedback mechanism in decoder blocks for progressive spatial refinement, (ii) a novel Order Statistics Layer (OSL) to capture extreme-value-based lesion details, and (iii) enhanced attention in skip connections to selectively emphasize medically relevant regions. These components collectively enable robust segmentation, particularly in low-contrast or heterogeneous Datasets. We evaluate AttenUNeT X on three benchmark dermoscopic Datasets International Skin Imaging Collaboration (ISIC) 2018, ISIC 2017, and Pedro Hispano Hospital Dataset (PH2), achieving superior segmentation performance across all. Our contributions lie in designing a clinically practical and technically novel segmentation framework, validated across diverse Datasets with strong generalizability for dermatology workflows. The overall workflow of the proposed AttenUNeT X framework, including preprocessing, feature extraction, feedback mechanism, and segmentation output, is illustrated in Fig. 1

## Literature survey

This section offers a thorough analysis of the many methods used to segment skin lesions in order to comprehend their working principles and pinpoint any possible drawbacks. Effective diagnostic techniques are becoming more and more important as the incidence of skin cancer grows worldwide. Accurately detecting and treating skin lesions, especially melanomas, may be extremely difficult. Manual evaluations by qualified dermatologists have been a major component of traditional diagnostic techniques, although they can be laborious and prone to inter-observer variability. Automated skin lesion segmentation systems have been created and put into use to overcome these inefficiencies. Recent technological developments, especially in the field of artificial-intelligence have significantly improved dermoscopic image analysis capabilities. In medical image processing, deep learning (DL) approaches have proven very useful, allowing for better skin lesion identification, segmentation, and classification.

These techniques make use of sophisticated neural network topologies, such U-Net, which is excellent at identifying the borders of lesions and identifying complicated patterns in dermoscopic images. By enabling the network to concentrate on important aspects of the images, the incorporation of attention mechanisms into these models enhances the segmentation process and raises the diagnostic accuracy. These cutting-edge methods greatly increase the possibility of early identification and better patient outcomes in the treatment of skin cancer. Conventional techniques for segmenting skin lesions have mostly depended on the extraction and detection of min-level features. The accuracy of segmentation has been further improved by advancements in feature aggregation. To improve global context extraction, the segmentation Transfers (SETR)^1^ modified the transformer for systematic segmentation and usd it in an encoder-decoder architecture. Ali et al.^2^ Proposes a two-stage SR pipeline combining SwinIR (ViT) and Diffusion Models to enhance and super-resolve RS images. SwinIR captures global context, while iterative DM restores fine details.

Mahmood et al.^3,4^ introduces an AI-enhanced microscopy framework for breast cancer diagnosis using image classification techniques. It employs Convolutional Neural Network (CNN) with transfer learning to classify breast histopathological images and demonstrates high precision in detecting cancer subtypes and emphasizes the need for explainable AI in medical microscopy applications. And also explore multi-modal neuroimaging approaches (MRI, PET, fMRI) combined with deep learning for early detection of Alzheimer’s disease. Highlights the effectiveness of 3D-CNN, hybrid CNN-LSTM models, and fusion architectures in integrating structural and functional imaging, It calls attention to real-time diagnosis, data imbalance, and clinical interpretability. As well as combines radiomics and deep learning (SE-ResNet152, VGGNet, CNN + LSTM) with swarm optimization for feature selection and classification. Uses Grad-CAM for interpretability achieves Area under curve (AUC) of 0.99 on mammography data in another proposal. Again the author Mahmood et al.^3,4^ proposes a deep learning approach to detect black fungus from CT and MRI scans using CNN-based architectures. The model achieves strong segmentation and classification performance. The work is relevant in post-COVID diagnostic contexts and focuses on early triaging and intervention. Hassan et al.^5,6^ introduce an attention-based deep neural network model for detecting dysarthria in speech data from patients with cerebral palsy and ALS. Using MFCC and log-Mel features, their model outperforms baseline CNNs. The study highlights how attention layers improve classification of subtle speech impairments. Mahmood et al.^7,8^ present MFF-HistoNet, a novel multiscale feature fusion network that integrates shallow and deep features from histopathological images. The model is designed for high sensitivity and accuracy in cancer subtype classification. Performance is benchmarked on public Datasets and shows superiority over conventional CNN models. Hassan et al.^5,6^ Proposes an ensemble of ResNet and Inception networks with GWO for feature selection. Yields high classification metrics (accuracy 96.5%, AUC 0.971) on WBCD and mammography Datasets.

Rehman et al.^9^ Uses Swin-ViT + DeepLabV3 + for segmentation and classification of kidney cancer in CT images. ASPP and Grad-CAM improve interpretability. Combines global and local features for tumor boundary delineation, Transformer topologies have recently shown remarkable results in the segmentation of skin lesions (Wu and Zhang^10^). Wang et al.^11,12^ efficiently address ambiguous boundary concerns, however this may miss important information between succeeding modules. There have also been suggestions for parallel adaptations in which the CNN and transformer properties are combined (Wu and Zhang^10^; Zhang et al.^13^). The investigation of rich multi-scale characteristics may be limited by these fusions, which frequently take place later in the transformer branch. With its primary classifications of edge detection with region growth techniques, image segmentation has undergone tremendous change. Whereas region growth divides the image based on local similarities, edge detection looks for discontinuities between areas. These techniques frequently have drawbacks, including poor segmentation accuracy, noise sensitivity, and the requirement for manual feature extraction, despite their fundamental function. Liu et al. highlighted the limitations of classical methods such as decision trees and SVMs, which require manual feature engineering, and emphasized the superiority of deep learning-based approaches. U-Net, with its encoder–decoder structure, automates feature extraction and improves segmentation accuracy, while attention mechanisms further enhance feature representation. Recent models, including MRP-UNet, have been shown to outperform traditional methods in segmentation tasks^14^. Ahmed et al. proposed DuaSkinSeg, a dual encoder model for skin cancer segmentation that integrates MobileNetV2 to capture local features and a ViT-CNN module to model global context. Experimental evaluation on ISIC Datasets demonstrated the model’s competitive performance compared with existing methods, highlighting its potential to improve segmentation accuracy and computational efficiency in dermatological diagnosis^15^.

Balraj et al. examined traditional and deep learning segmentation methods, providing a comparative analysis of their effectiveness in terms of Dice Similarity Coefficient (DSC) and Jaccard Index. Their proposed MADR-Net architecture, which incorporates multi-level attention and dilated residual connections, achieved superior performance and outperformed several state-of-the-art approaches^16^. Innani et al. developed Efficient-GAN, a generative adversarial network framework for skin lesion segmentation from dermoscopic images. The model uses a generator–discriminator structure to produce accurate lesion masks and outperformed existing methods across multiple evaluation metrics. Furthermore, a lightweight variant, Mobile-GAN, was introduced, offering comparable results with significantly fewer parameters, thereby facilitating deployment in resource-constrained environments^17,18^. Soni et al. addressed segmentation challenges caused by image variability and artifacts by proposing ARCUNet, which combines residual convolutions with attention mechanisms. The model achieved outstanding results on ISIC Datasets, reporting accuracies of 98.12%, 96.45%, and 98.19%, with Jaccard scores of 91.14%, 88.33%, and 93.53%, demonstrating its robustness and high precision in lesion segmentation^19^. Bai et al. introduced SSR-UNet, a novel U-shaped segmentation architecture that incorporates bidirectional scanning to enhance feature extraction. Additionally, a spatially constrained loss function was designed to stabilize gradients and improve overall training performance, leading to improved segmentation accuracy^20^. Yang et al. presented a deep learning model for skin scar segmentation using a dual encoder network that integrates CNN and Swin Transformer architectures. The approach introduced a multi-scale feature fusion module along with a novel multi-pooling channel–spatial attention mechanism. Experimental results showed strong performance, with 96.01% accuracy, 77.43% precision, 90.17% recall, 71.38% Jaccard Index, and 83.21% Dice Coefficient, underscoring its potential for precise scar analysis in clinical applications^21^. Focus-net, which focuses on lung lesion and melan2oma segmentation, was developed by Kaul et al.^22^ by combining the Squeeze as well as Excite module with ResNet.

In bio-medical research, deep learning has been extensively recognized for its use in pre-segmentation procedures for diagnostic tasks. Using the ISIC 2018 Dataset, Mahmud et al.^23^ Introduces SkinNet-14, a lightweight transformer-based model built on Compact Convolutional Transformer (CCT) to classify skin cancer from 32 × 32 pixel dermoscopy images. Incorporates optimized data augmentation and preprocessing to handle class imbalance and noise while significantly reducing training time, making it suitable for resource-limited clinical settings. Li et al.^24^ Proposes a U-Net-based architecture that integrates multiscale input fusion with Res2SE and pyramid dilated convolution modules for improved skin lesion segmentation. Res2SE captures fine-grained semantic and channel features, while the pyramid structure enhances multiscale context awareness. Achieves strong performance on ISIC Datasets with better boundary localization and robustness to lesion size variation. A detailed comparative summary of existing skin-lesion segmentation approaches is provided in Table 1.

**Table 1:** summary of existing skin-lesion segmentation approaches.

Study	Metrics	Dataset	Methodology	Application
Wang and Xu^11^	Accuracy: 95.2%, AUC: 91.0%	ISIC 2018	Boundary-Aware Method	Skin lesion segmentation
Wu and Zhang^10^	Accuracy: 96.0%,	ISIC, PH2	Comprehensive Review	Skin lesion segmentation methods
Dai et al.^25^	Accuracy: 94.5%, Precision: 92.1%, IoU: 87.2%	ISIC 2018	Multi-Scale Residual Encoding and Decoding (Ms RED)	Skin lesion segmentation
Ta et al.^26^	Accuracy: 95.3%, F1 Score: 94.0%	Private Dataset	Complementary and Contrastive Network	Stimulus segmentation and generalization
Deng^27^	AUC: 92.7%, IoU: 89.1%	ISIC	FAT-Net (Feature Adaptive Transformers)	Automated skin lesion segmentation
Jalil and Usman^28^	Accuracy: 93.7%, F1 Score: 92.2%	ISIC 2018 (Task 3)	Improved Tuna Swarm-based U-EfficientNet	Skin lesion segmentation
Innani et al.^17,18^	F1 Score: 90.5%, IoU: 85.6%	PH2, ISIC 2018, ISIC 2017	Generative Adversarial Networks (GANs)	Skin lesion segmentation
Dinesh abd Lakshmanan^29^	Dice: 0.908	Private Dataset	DeepOverlay L-U-Net	Hybrid learning for lesion detection
Li et al.^24^	Dice Similarity Coefficient (DSC): 92.13% (ISIC 2018), 90.62% (ISIC 2017), 88.74% (ISIC 2016)	ISIC 2016, ISIC 2017, ISIC 2018 Dataset	Enhanced U-Net with Multiscale input fusion Res2SE blocks Pyramid dilated convolution for multiscale context	Skin lesion segmentation
Liu et al.^30^	Accuracy: 95.51%; Sen: 88.57%; Spec: 93.44%; Jaccard: 91.28%; Dice: 92.36	ISIC 2016, ISIC 2017, ISIC 2018, PH2, and Human Against Machine 10,000 Dataset (HAM10000 Datasets)	MRP-UNet for skin lesion segmentation: U-Net with attention mechanisms; automated feature extraction; improved encoder-decoder structure	Skin lesion segmentation
Ahmed et al.^15^	Accuracy: 97.08%; Precision: 92.44%; Recall: 91.12%; Jaccard: 84.63%; F1Score: 91.78	ISIC Datasets	Dual encoder: MobileNetV2 (local features) + ViT-CNN (global context)	Skin lesion segmentation
Balraj et al.^16^	Accuracy: 96.36%; Precision: 92.50%; Recall: 87.35%; Jaccard: 81.44%; Dice: 89.46	dermoscopy images, (ISIC) in 2017 Datasets	MADR-Net:Comparison of traditional & DL approaches; DSC & Jaccard analysis	Skin lesion segmentation
Innani et al.^17,18^	Accuracy: 94.5%; Spec: 95.5%; Sen: 93.6%; Jaccard: 83.6%; Dice: 90.1	dermoscopic images	Efficient-GAN / Mobile-GAN: GAN-based segmentation; generator + discriminator; lightweight Mobile-GAN	Skin lesion segmentation
Soni et al.^19^	Accuracy: 98.19%; Jaccard: 93.53%; Dice: 96.88	ISIC Datasets	ARCUNet : Residual convolutions + attention mechanisms	Skin lesion segmentation
Bai et al.^20^	Accuracy: 95.34%; Spec: 97.54%; Sen: 88.49%; Jaccard: 71.38%; Dice: 90.21	ISIC Dataset, PH2	SSR-UNet: U-shaped segmentation with bidirectional scanning + spatially-constrained loss	Skin lesion segmentation
Yang et al.^21^	Accuracy: 96.01%; Precision: 77.43%; Recall: 90.17%; Jaccard: 71.38%; Dice: 83.21	Skin scar Dataset	Dual Encoder (CNN + Swin Transformer) Multi-scale feature fusion + multi-pooling channel-spatial attention	Skin lesion segmentation

## Data set description

### ISIC 2018 dataset

This study utilized three widely accepted dermoscopic image Datasets for training, validation, testing, and cross-validation: ISIC 2018, PH2 and ISIC 2017**.** ISIC 2018 (Task 3: Lesion Segmentation) was the primary Dataset used for training and validation due to its large size and diversity. It consists of 2594 expertly annotated dermoscopic images spanning multiple lesion types. In our setup, 1815 images were used for training, 259 for validation, and 520 for testing. This Dataset provides a rich variety of skin lesion morphologies and lighting conditions, making it suitable for training deep learning models.

### PH2 dataset

The PH2 Dataset consists of 200 dermoscopic images with corresponding ground-truth masks. PH2 was not used in the main training pipeline, but you experimented with it by splitting it into training/validation for comparative analysis. For supplementary analysis, the PH2 dataset (200 images) was split into 80% training (160 images) and 20% validation (40 images). Small-split experiment to check model adaptability on a limited dataset (hence PH2 appears in Table 5). This split was used only to test the adaptability of the proposed model on limited data, as PH2 is too small to serve as a primary training dataset.

The full PH2 dataset was also used separately as an external test set (Table 10) to evaluate cross-dataset generalization and complete dataset as an external test set to evaluate generalization (results reported in Table 10). This dual usage was only for experimental comparison and does not affect the main training pipeline, which is based on ISIC 2018.

###  ISIC 2017 dataset

ISIC 2017, which includes over 2,000 annotated dermoscopic images, was utilized for cross-validation purposes only. Specifically, we allocated 1400 images for training, 300 for validation, and 300 for testing, solely to evaluate the transferability and domain adaptability of the proposed model trained on ISIC 2017.

To mitigate overfitting risks especially when testing on smaller Datasets like PH2 we incorporated several measures:

- Extensive data augmentation (rotation, flipping, zooming, and shearing)
- Use of regularization techniques such as dropout and early stopping
- Architecture-level enhancements that encourage spatial feature generalization**,** such as feedback loops and attention modules

These strategies collectively enabled the model to generalize well across Datasets of varying size and complexity. The dataset split configuration used for training, validation, and testing is summarized in Table 2.

**Table 2:** Datasets splitting for training, validation, testing in skin lesion segmentation.

Dataset	Total images	Training images	Validation images	Testing images
ISIC 2018	2594	1815	259	520
PH2	200	160	40	200 (External testing)
ISIC 2017	2000	1400	300	300

## Methodology

The proposed AttenUNeT X model builds upon the classic U-Net architecture, incorporating three significant enhancements tailored for precise and robust skin lesion segmentation**:** Feedback Mechanism in Decoder Blocks, Order Statistics Layer (OSL), and Enhanced Attention Modules. These components are strategically designed to improve boundary delineation, capture extreme-value features in lesion texture, and highlight diagnostically relevant regions all of which are critical in dermatology. In preparing our model for skin lesion segmentation, we embarked on a journey utilizing two foundational Datasets: ISIC and PH2. These Datasets offered a diverse array of dermoscopic images, crucial for training our model to adeptly handle the various features of skin lesions. To ensure uniformity in the preparation of images and segmentation masks, we developed a function called *load_and_preprocess_Dataset*. This function serves as a comprehensive pipeline, meticulously designed to load, preprocess, and augment the Dataset. The ultimate goal was to guarantee that our model was trained on consistent and high-quality inputs.

###  Pre-preprocessing

#### Black hat transformation for hair detection

In our preprocessing pipeline, the initial step involved employing a black hat transformation to isolate hair in the images. This morphological operation emphasizes darker regions, thereby facilitating the differentiation of hair from the lighter surrounding skin. The mathematical definition of the black hat transformation is expressed as:

1 $${\text{Black}}\,{\text{Hat}} = f_{{{\text{closing}}}} \left( I \right) - {\text{I}}$$

where ${\text{f}}_{\text{closing }}\left(\text{I}\right)$ denotes the morphological closing of the input image I using a $17 \times 17$ rectangular structuring element.

#### Binary thresholding

Following the black hat transformation, we applied binary thresholding to create a hair mask. This step sharpens the definition of detected hair regions by setting a threshold T of 50. The thresholding process can be represented mathematically as:

2 $$p\left( {x,y} \right) = \left\{ {\begin{array}{*{20}l} {255} \hfill & {{\text{if}}\,{\text{I}}\left( {x,y} \right)> {\text{T}}} \hfill \\ 0 \hfill & {{\text{otherwise}}} \hfill \\ \end{array} } \right.$$

where $\text{I}\left(\text{x},\text{y}\right)$ denotes the intensity at each pixel.

#### In-painting for hair removal

With the hair regions highlighted in a binary mask, we performed in-painting to seamlessly fill these areas. Utilizing the Telea in-painting method, we reconstructed the identified regions by propagating pixel values from neighboring areas, effectively “healing” the image while preserving the integrity of the lesions.

#### Resizing and normalization

To ensure uniformity across all samples, we resized each image to a standardized dimension of 256 × 256 pixels. Subsequently, we normalized the pixel dimension values to the between [0, 1] using the formula:

3 $$\text{Normalized Pixel Value}=\frac{\text{Original Pixel Value}}{255}$$

This normalization step maintains consistent brightness and contrast across images, allowing the model to concentrate on lesion features rather than variations in lighting or imaging conditions across the Datasets.

#### Data augmentation

To further enhance our Dataset, we integrated ImageDataGenerator for data augmentation. Given the high variability of lesion appearances in the Datasets, augmentation proved vital in helping our model generalize effectively by exposing it to diverse transformations. The configurations we implemented included:

- Rotation: arbitrary rotations within ± 30°.
- Width: arbitrary horizontal move within 10% of the entire width.
- Height: arbitrary vertical move within 10% of the entire height.
- Shearing: A shearing sort of 0.2.
- Zooming: Zoom transformations up to 40%.
- Flipping: Both horizontal and vertical flips applied randomly.

**Fig. 2:**
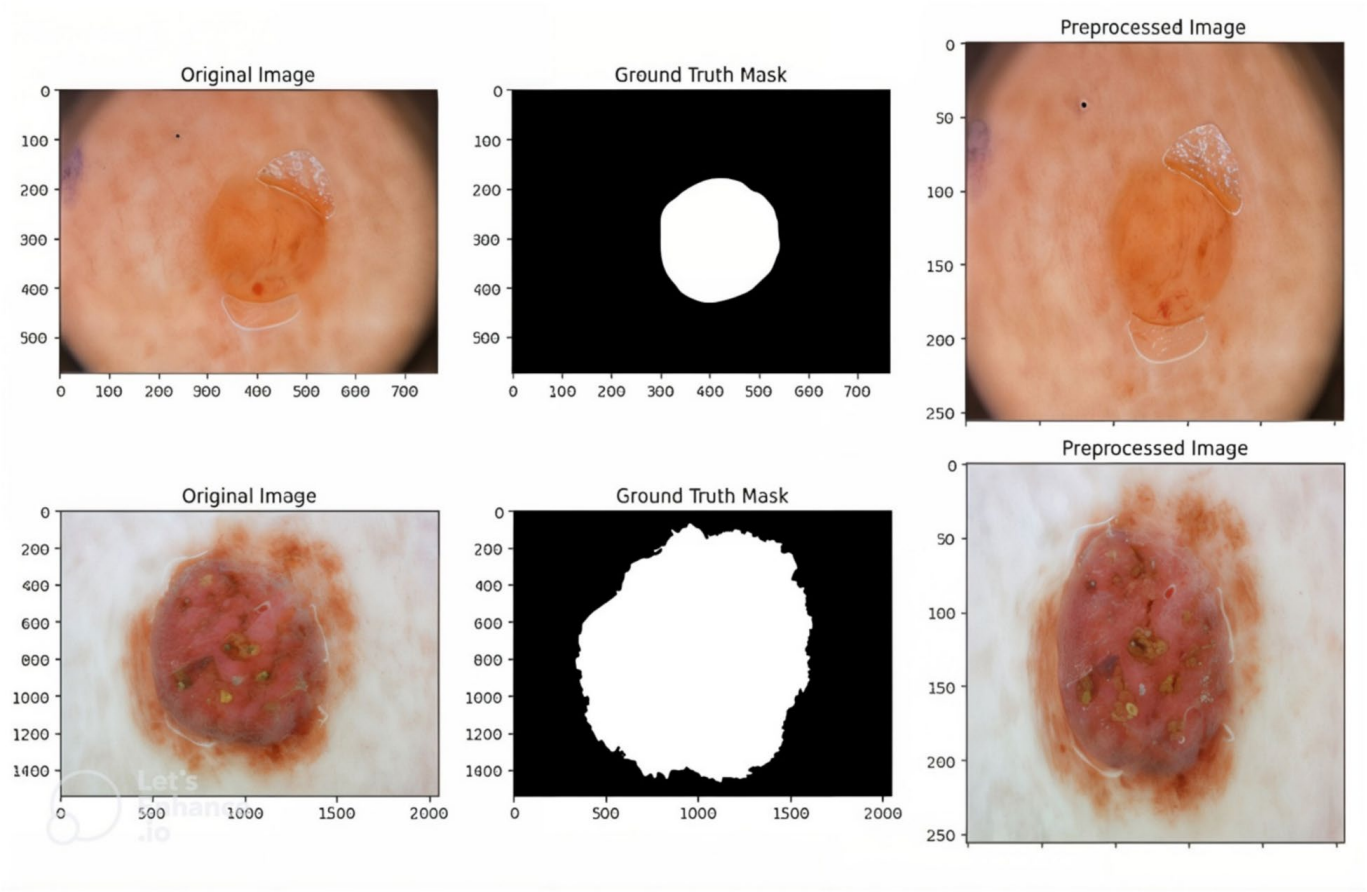
Preprocessing steps performed on the dataset. Original dermoscopic image, corresponding lesion mask aligned for training and hair-removed and resized image.

These augmentations introduced spatial and orientation-based variations, enriching the learning experience and mitigating over-fitting. By simulating a variety of real-world conditions, our model learned to recognize lesions with greater robustness. These steps standardize lesion input and simulate real-world variability, improving generalization across Datasets. While the visual appearance of the preprocessed image remains similar to the original, significant pixel-level transformations occur during preprocessing. Techniques like Black Hat transformation and in-painting do not drastically alter the RGB values but remove subtle obstructions such as hairs, which are difficult for a model to ignore. Additionally, resizing, normalization, and augmentation ensure that all images are uniform and model-friendly. These improvements are not always apparent to the human eye, but they play a critical role in improving feature extraction and segmentation accuracy. Preprocessed examples of ISIC 2018 dermoscopic images after hair removal and augmentation are shown in Fig. 2.

### Architecture

As we embarked on our journey to refine the U-Net structural design, we recognized the importance of crafting a robust framework tailored to intricacies of lesion segmentation. We included two essential elements: a feedback mechanism embedded in the decoder blocks and an Ordered Statistics Layer for improved feature extraction. Our architecture is based on a carefully crafted U-Net model that can process 256 × 256 pixel input images. The structure is revealed through a number of thoughtfully built blocks. AttenUNeT X follows the standard encoder**–**decoder structure of U-Net with key architectural improvements.

#### Encoder

Sequential convolutional blocks extract features at multiple scales, activated via ReLU, and downsampled via max pooling. This helps the model capture hierarchical features of lesion texture and color. Two primary components make up the encoder, which is intended to gradually extract information from the input image. The ReLU function activates the convolutional layers used in each block. A max-pool layer that deliberately lowers dimensionality complements the convolutional layers with 32, 3 × 3 filters in the first encoder block. The model ability to take out pertinent skin tone is further improved in its second encoder block, when the number of filters increases to 64.

#### Order statistics layer

Positioned at the bottleneck, the OSL computes normalized maximum and minimum values across feature map channels and concatenates them to the feature representation. Lesions often show extreme intensities at their borders or cores. Capturing this distribution enhances the model’s ability to discriminate between lesion and background, particularly in low-contrast areas. We realized that a reliable way to extract and represent important characteristics from the input images was essential in our efforts to improve the design of U-Net for skin lesion segmentation. To fill this gap, we created the Order Statistic Layer, a brand-new element made especially for getting extreme values from the feature maps produced by the layers that use convolution. These extreme values might be either minimum or maximum. In order to properly define skin lesions, our model must be able to concentrate on both obvious characteristics and minute details, which is greatly aided by this layer. The inclusion of the Order Statistics Layer (OSL) is particularly beneficial in handling low-contrast lesions, where boundaries often blend into surrounding skin. Unlike global preprocessing techniques such as histogram equalization or contrast normalization, which apply uniform corrections across the entire image, OSL functions as a learnable and adaptive layer. This allows the network to dynamically capture local intensity distributions during training, thereby enhancing fine-grained boundary delineation in challenging cases.

#### Input tensor definition

With dimensions H × W × C, the input tensorX is where the process starts, where:

- $H$ is the feature map’s height.
- $W$ stands for the feature map’ width.
- $C$ is the feature map generated from the preceding convolutional layers has a channel count.

This tensor is a crucial tool for the segmentation process as it contains extensive information on the characteristics that were taken from the input image.

*Calculation of Minimum and Maximum Values:* In order to effectively collect the highest and lowest values that are important for the representation of features, we calculate the Optimized max and min thresholds throughout the contribution tensor’s channel dimension. These operations’ formulas are:$H\times W\times C$

4 $${\text{small}}_{{{\text{order}}_{{\text{k}}} }} = \frac{1}{k}\sum _{{{\text{i}} = 1}}^{{\text{k}}} {\text{min}}_{i} \left( {{\text{X}},{\text{ axis}} = - 1,{\text{ keepdims}} = {\text{True}}} \right)$$

5 $${\text{large}}\_{\text{order}}_{k} = \frac{1}{{\text{k}}}\sum _{{{\text{i}} = 1}}^{{\text{k}}} {\text{max}}_{i} \left( {{\text{X}},{\text{ axis}} = - 1,{\text{ keepdims}} = {\text{True}}} \right)$$

In addition, we scale the variation and lessen the susceptibility to outliers by normalizing these values:

6 $${\text{small}}\_{\text{order}}_{{{\text{norm}}}} = \frac{{{\text{small}}\_{\text{order}}}}{{{\text{large}}\_{\text{order}} + \in }}$$

7 $${\text{large}}\_{\text{order}}_{{{\text{norm}}}} = \frac{{{\text{large}}\_{\text{order}}}}{{{\text{large}}\_{\text{order}} + \in }}$$

Here:

- $small{\_\text{order}}_{norm}$ and $large{\_\text{order}}_{norm}$ are the normalized minimum and maximum values.
- $\in$ is a small constant to avoid division by zero.
- $k$ refers to the number of iterations used to compute the minimum and maximum values

The model may concentrate on both the lowest along with highest feature values, that might be important markers for lesion features, according to these calculations.

*Combining Statistics with the feature map:* Following the extraction of extreme values, we concatenate these statistics with the novel feature map X to augment the attribute representation. The concatenation operation is represented mathematically as:

8 $${\text{X}}_{{{\text{enhanced}}}} = {\text{Concat}}({\text{X}},{\text{ small}}\_{\text{order}},{\text{large}}\_{\text{order}}_{{{\text{norm}}}} )$$

As a result, the new tensor ${\text{X}}_{\text{enhanced}}$ ​ possesses dimensions $\text{H}\times \text{W}\times (\text{C}+2)$, effectively incorporating the minimum and maximum values as additional channels. This augmentation enriches the feature space, providing the model with more comprehensive information for the segmentation task.

#### Impact on learning

The integration of the extreme values into the feature representation significantly enhances the model’s learning capabilities. By emphasizing critical features, the layer facilitates a more nuanced understanding of the data. The output from the feature extraction process can be described as:

9 $${\text{X}}_{{{\text{final}}}} = {\text{FeatureExatractor}}({\text{X}}_{{{\text{enhanced}}}} )$$

In this equation, $\text{FeatureExatractor}$ symbolizes the subsequent layers (typically convolutional layers) that further process the enhanced feature map to extract relevant patterns for segmentation.

#### Facilitating robust feature learning

By capturing the extreme statistics, the Order Statistics Layer empowers the model to learn effectively from both global and local feature characteristics. The final segmentation output can be represented as:

10 $${\text{Y}}_{{{\text{OUTPUT}}}} = \sigma ({\text{W}}_{{\text{f}}} *{\text{X}}_{{{\text{final}}}} + {\text{b}}_{{\text{f}}} )$$

where:

- ${\text{Y}}_{\text{OUTPUT}}$ is the final segmentation mask.
- ${\text{W}}_{\text{f}}$ is the weight matrix associated with the final convolutional layer.
- ${\text{b}}_{\text{f}}$​ represents the bias term.
- σ is the sigmoid activation function, which produces pixel values in the range [0,1].

This process ensures that the model is capable of generating precise segmentation masks, crucial for accurate medical diagnoses. Lesions often show extreme intensities at their borders or cores. Capturing this distribution enhances the model’s ability to discriminate between lesion and background, particularly in low-contrast areas. In the architecture, the Bottleneck Block is a crucial intersection. Here, we use 128-filter convolutional layers, which enable the model to better explore the intricacies of the given input. This block is essential for making the switch from encoder into decoder easier.

#### Decoder with feedback mechanism

We incorporate an upsampling layer and concatenate it with matching encoder features as we move up through the Decoder Blocks. As we recreate the image, this important phase guarantees that important information is maintained. To preserve the model’s nonlinear properties, the decoder uses convolutional layers using 64 and 32 filters and applies the same ReLU activation.

The feedback mechanism contributes to iterative refinement by reintroducing decoder features into earlier layers, allowing progressive error correction in lesion boundary prediction. Unlike attention-guided mechanisms, which emphasize salient features within a single forward pass, our feedback design enables multi-pass refinement without requiring multiple networks. Compared to multi-stage architectures, which often rely on cascaded heavy models, our feedback module provides a lightweight and computationally efficient solution for fine boundary delineation. Each decoder block performs: Upsampling, Skip connection from corresponding encoder layer, Feedback connection from earlier decoder outputs. As we ventured into the decoder blocks, we recognized the need for a feedback mechanism to reinforce information flow through recurrent connections. This innovative approach involves concatenating outputs from earlier layers, followed by convolutional operations and batch normalization:

11 $${\text{Y}} = {\text{Concat}}\left( {{\text{X}},{\text{S}}} \right)$$

where $\text{S}$ represents the skip connection from the encoder.

Next, we apply convolutions:

12 $${\text{Y}}_{1} = {\text{ReLU}}({\text{BatchNorm}}\left( {{\text{W}}_{1} {\text{*Y}}} \right)$$

13 $${\text{Y}}_{2} = {\text{ReLU}}({\text{BatchNorm}}({\text{W}}_{2} *{\text{Y}})$$

Finally, we obtain the output:

14 $${\text{Y}}_{{{\text{output}}}} = ({\text{W}}_{{\text{f}}} *{\text{Y}}_{2} )$$

This feedback mechanism iteratively improves details at each level of decoding, successfully capturing lesion aspects.

#### Attention modules

Enhanced spatial attention is integrated into the encoder-decoder skip connections. This allows the network to focus on key lesion areas while suppressing background noise. We created a U-Net model with a mechanism for attention through integrating the feedback enabled decoding blocks with the Order Statistics Layer. This setup enables our model to effectively reject irrelevant input while concentrating its learning on key traits. The function known as the sigmoid activation drives the output layer’s creation of segmentation masks, which have pixel values between 0 and 1.

**Fig. 3:**
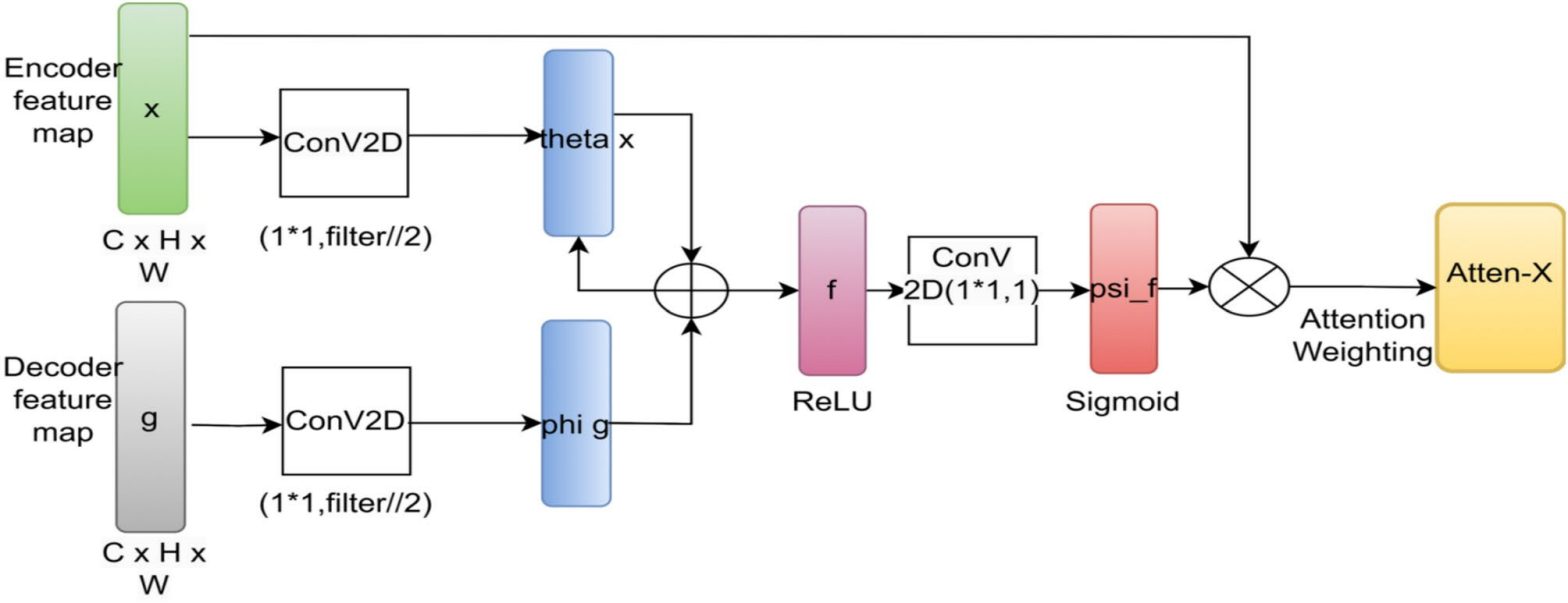
Architecture of the attention module used in AttenUNeT X. The block performs spatial and channel attention before merging features in the decoder.

Essentially, this architectural development makes it possible for our model to produce precise and trustworthy segmentation masks for intricate skin lesions, increasing its versatility across the PH2 and ISIC Datasets. By making these improvements, we want to expand our deep learning model’s potential and keep it at the very forefront of segmenting skin lesions in technology. Traditional U-Net decoders reconstruct segmentation maps in a one-pass, top-down manner. Our feedback mechanism allows iterative refinement of lesion boundaries by reusing earlier decoding outputs. This is especially helpful in recovering fine details and irregular borders of lesions. Here, θ(x) and φ(g) denote learnable linear projections generating query and key embeddings used for attention weighting within the skip pathway. Often share appearance with surrounding skin. Attention mechanisms enable the model to differentiate contextually important features like asymmetry, pigment spread. The schematic representation of the enhanced attention block used in AttenUNeT X is provided in Fig. 3.

#### Output layer

**Fig. 4:**
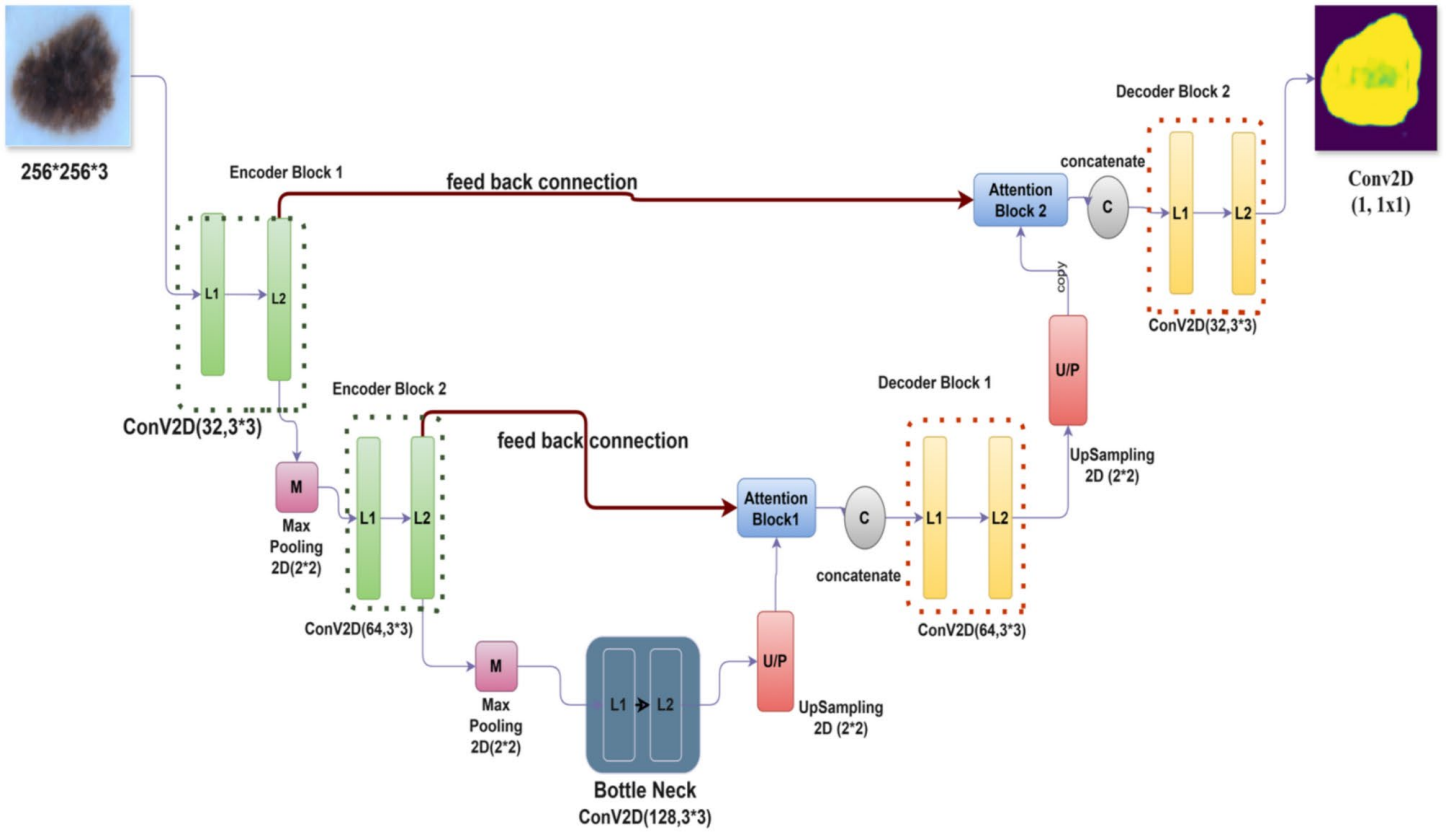
Detailed architecture of the proposed AttenUNeT X model, integrating encoder–decoder blocks with Order Statistics Layer (OSL), iterative feedback mechanisms, and attention modules for lesion boundary refinement. Each layer’s filter size and skip connections are indicated. BN—Batch Normalization; ReLU—Rectified Linear Unit.

A final 1 × 1 convolution followed by a Sigmoid activation produces the segmentation mask, with pixel-level probabilities indicating lesion presence. The output layer appears, which uses a convolution layer using a single 1 × 1 filter. Our segmentation masks are created by this layer using a Sigmoid activation function, which turns the complex data into useful insights. Although the visual representation in Fig. 4 may appear dense, each module follows a consistent labeling convention. Encoder blocks (green) consist of two convolutional layers (L1 and L2), followed by max pooling (M) to reduce spatial dimensions. The central bottleneck layer (blue) increases feature depth and is followed by two decoder blocks (yellow), which incorporate upsampling (U/P) and concatenation (C) with the corresponding encoder outputs. Attention blocks are inserted before each decoder block to focus on important lesion features. Importantly, feedback connections are introduced from decoder to encoder stages to enable reactivation of relevant features. All Conv2D operations are annotated with their respective parameters (filters, kernel size), and the architecture is designed to maintain symmetry while enhancing lesion detail learning. The detailed architecture integrating encoder–decoder blocks with Order Statistics Layer, iterative feedback, and attention modules is presented in Fig. 4.

**Algorithm:**
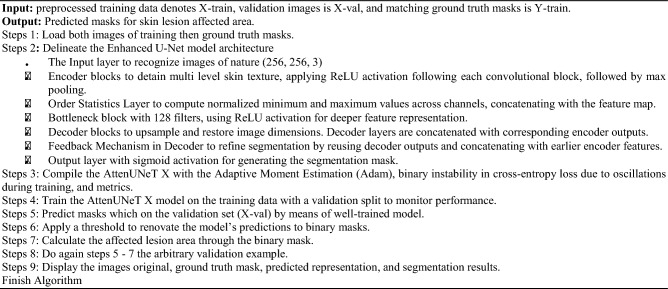
Skin lesion segmentation using AttenUNeT X

### Training setup

The AttenUNeT X model was trained using the following hyperparameters:

- Optimizer**:** Adam optimizer with β1 = 0.9 and β2 = 0.999
- Learning rate(LR)**:** 1e-4 with a ReduceLROnPlateau scheduler (factor 0.1, patience = 10)
- Batch size: 16
- Maximum epochs: 150
- Dropout rate: 0.3 applied in decoder blocks
- Loss function: A hybrid loss combining Dice loss and Binary Cross-Entropy (BCE) loss
- Early stopping criterion: Training was stopped if validation loss did not improve for 15 consecutive epochs

These hyperparameter choices were determined empirically to balance segmentation accuracy and training stability.

## Experimental results

### Quantitative results

The performance of AttenUNeT X was evaluated on three Datasets: ISIC 2018, PH2, and ISIC 2017**.** Metrics reported include Dice Coefficient, Intersection over Union (IoU), and pixel-level accuracy. As shown in Table 3, the model achieved a Dice of 0.9211, IoU of 0.8533, and accuracy of 0.9824 on the ISIC 2018 test set. Similar performance was observed on PH2 (Dice: 0.9500, IoU: 0.9000) and ISIC 2017 (Dice: 0.8542, IoU: 0.7454), demonstrating robust generalization.

**Table 3:** Ablation study on ISIC 2018 Dataset showing incremental contributions of attention, feedback, and OSL modules to segmentation performance.

Configuration	Dice coefficient	IoU	Accuracy
Baseline U-Net	0.8793	0.7995	0.9632
+ Attention Only	0.8946	0.8204	0.9705
+ OSL Only	0.9017	0.8293	0.9738
+ Feedback Only	0.9084	0.8362	0.9776
Full AttenUNeT X	0.9211	0.8533	0.9824

### Ablation study

To validate the individual contributions of each proposed component, we conducted an ablation study on the ISIC 2018 validation set. The results are summarized in Table 3. Each configuration builds incrementally toward the full AttenUNeT X model. The dataset split configuration used for training, validation, and testing is summarized in Table 2.

Table 3 Ablation study on ISIC 2018 Dataset showing incremental contributions of attention, feedback, and OSL modules to segmentation performance.

These results show that each module contributes progressively to performance. The feedback mechanism provided the largest boost in boundary accuracy, while OSL helped with discriminating fine lesion textures. Their combination leads to superior segmentation quality.

This part, we present the experimental results obtained from our study using the proposed AttenUNeT X model for skin lesion segmentation. The model’s performance was evaluated on three distinct Datasets: the PH2 Dataset, and the ISIC 2018 Dataset, ISIC 2017 Dataset. Each Dataset provided unique challenges and valuable imminent into the segmentation capabilities for skin lesions.

### Performance metrics

To review the segmentation feat, we employed several key metrics:

1. *DiceCo-efficient (DC)* This statistic calculates how much the ground truth and the anticipated segmentation mask overlap. It’s described as below, where the sets of anticipated pixels are denoted by A and the ground truth pixels by B.
  15 $${\text{DC}} = \frac{{2\left| {{\text{A}} \cap {\text{B}}} \right|}}{{\left| {\text{A}} \right| + \left| {\text{B}} \right|}}$$
2. *Intersection over Union (IoU)* sometimes referred to as the Jaccard’s Index, determines the segmentation accuracy by dividing the juncture of the ground-truth and predicted pixels by their combination are below, Where the sets of anticipated pixels are denoted by A and the ground truth pixels by B.
  16 $${\text{IoU}} = \frac{{\left| {{\text{A}} \cap {\text{B}}} \right|}}{{\left| {\text{A}} \right| \cup \left| {\text{B}} \right|}} = \frac{{{\text{DC}}}}{{2 - {\text{DC}}}}$$
3. *Pixel accuracy (PA)* Out of all the pixels in the image, this measure shows the percentage of pixels that were properly classified:
  17 $$\text{PA}=\frac{\text{No}.\text{ of Correct Predicts}}{\text{Total No}.\text{ of Predicts}}$$
4. *Precision* which indicates the ratio of true + ^ve^ predictions among all + ^ve^ predictions:
  18 $$\text{Precision }=\frac{\text{True Positives }}{\text{True Positive}+\text{False Positive}}$$
5. *Recall* This metric represents the proportion of true positive predictions among all actual positives:
  19 $$\text{Recall }=\frac{\text{True Positives }}{\text{True Positive}+\text{False Negative}}$$
6. *Area under curve (AUC)* AUC measures a binary classification model’s overall effectiveness. AUC values were computed from Receiver Operating Characteristic (ROC) analysis using the scikit-learn library; ROC curves are not shown due to space limitations, but values were derived directly from these plots.

### ISIC 2018 dataset (Task 3)

**Fig. 5:**
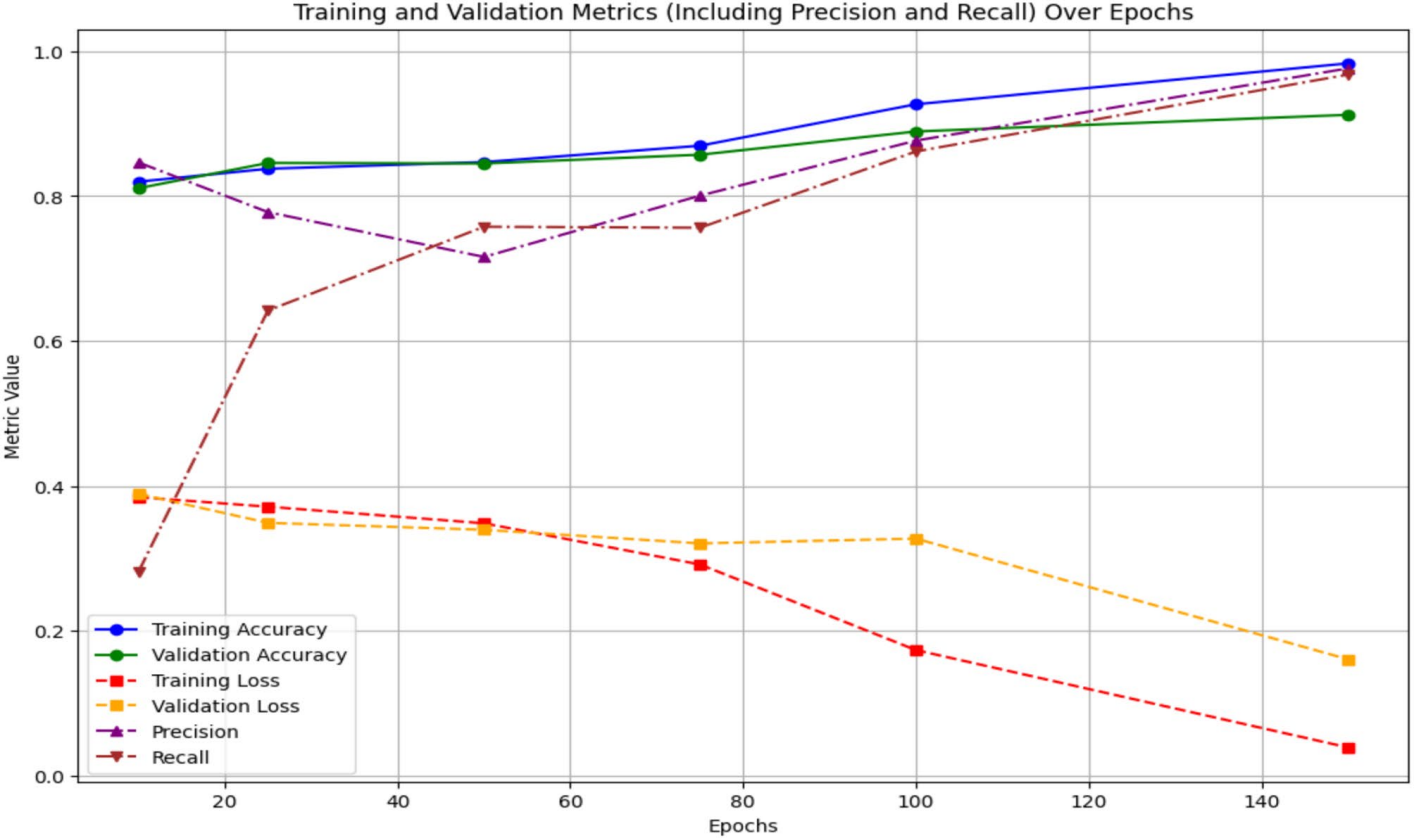
Results of ISIC 2018 Dataset training and validation metrics over 150 epochs.

The model shows significant gains in all important measures as training goes on through 10 to 150 epochs. With a comparable drop in training loss between 0.3846 to 0.0388, training accuracy rises from 0.8199 to 0.9834. Although validation loss varies significantly, suggesting possible over-fitting, validation accuracy also increases, reaching 0.9123 after 150 epochs. Both recall and precision exhibit consistent rises, reaching their highest points at 150 epochs with 0.9765 and 0.9679, respectively. On using ISIC 2018 Dataset, these patterns show good learning and performance. Training and validation performance across epochs for ISIC 2018 are summarized in Table 4. Training and validation accuracy–loss curves for the ISIC 2018 dataset are displayed in Fig. 5.

**Table 4:** Training and validation performance across epochs on ISIC 2018 Dataset.

Epochs	Training ACC	Training loss	Val-ACC	Val-loss	Precision’s	Recall’s
10	0.8199	0.3846	0.8109	0.3892	0.8466	0.2809
25	0.8378	0.3714	0.8458	0.3491	0.7779	0.6419
50	0.8470	0.3485	0.8451	0.3395	0.7160	0.7577
75	0.8696	0.2916	0.8572	0.3207	0.8006	0.7563
100	0.9269	0.1733	0.8893	0.3273	0.8767	0.8617
150	0.9824	0.0388	0.9123	0.1606	0.9765	0.9679

### PH2 dataset

**Fig. 6:**
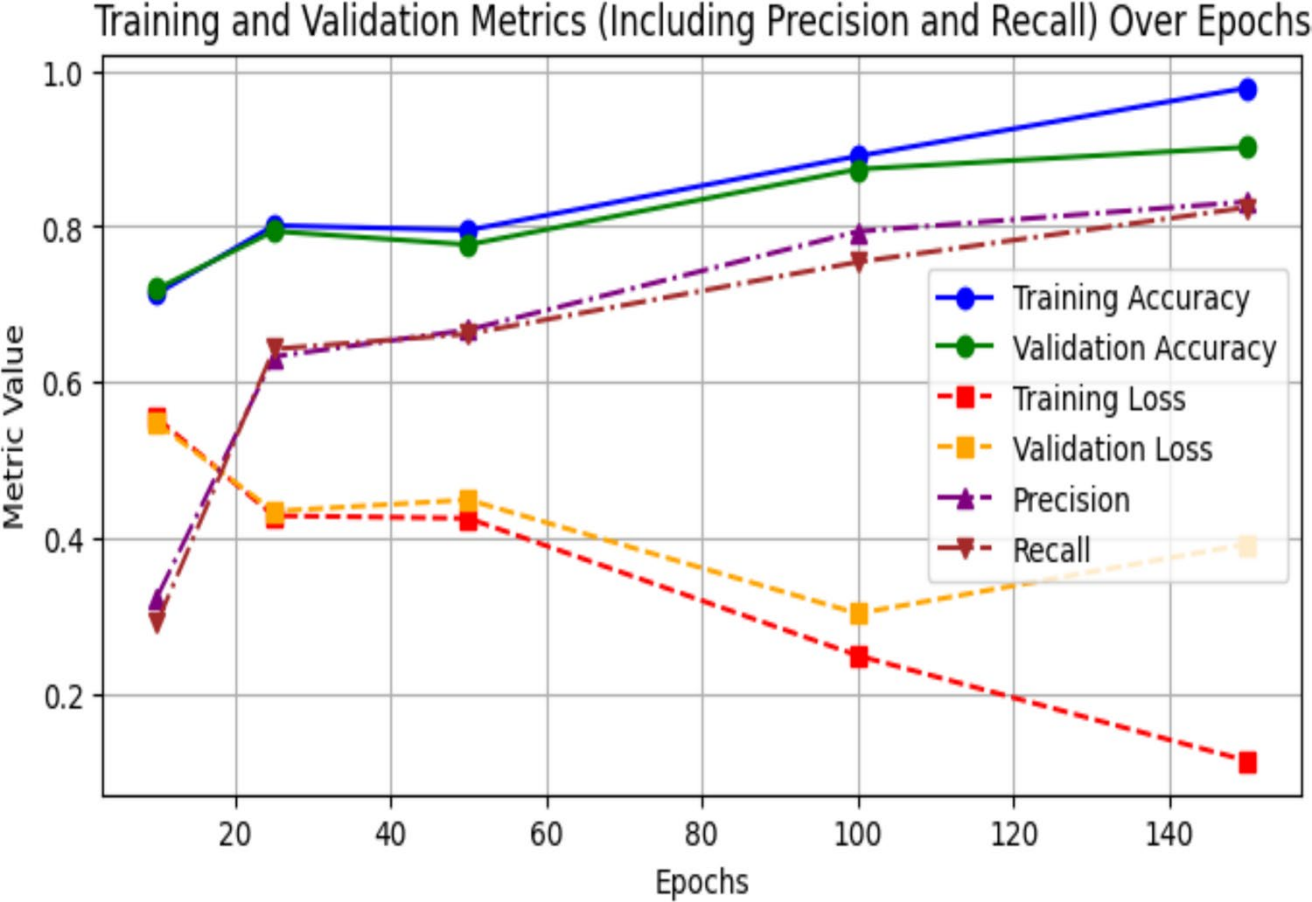
Results of PH2 Dataset training and validation metrics over 150 epochs.

The model shows consistent improvement with further training for the PH2 Dataset. Training loss dramatically decreases via 0.3846 to 0.1086, indicating successful learning, while training accuracy increases from 0.8256 over 10 epochs reaching 0.9563 at 150 epochs. Similar trends are shown in validation accuracy, which reaches 0.9023 by 150 epochs. However, validation loss varies, suggesting some over fitting in subsequent epochs. With improvements of 0.9237 and 0.9290 after 150 epochs, respectively, precision and recall also show promising segmentation performance. With further training, these findings validate the model’s ability to detect skin lesions properly and its resilience on the PH2 Dataset. The epoch-wise training and validation results for PH2 dataset are detailed in Table 5. Performance trends across training epochs for the PH2 dataset are plotted in Fig. 6.

**Table 5:** Training and validation performance across epochs on PH2 Dataset.

epochs	Training ACC	Training loss	Val—ACC	Val—loss	Precision’s	Recall’s
10	0.8256	0.3846	0.8339	0.3700	0.6914	0.7547
25	0.8453	0.3338	0.8486	0.3366	0.7177	0.7744
50	0.8607	0.3129	0.8606	0.3119	0.7611	0.7538
75	0.8835	0.2694	0.8632	0.3187	0.8219	0.7226
100	0.8980	0.2426	0.8661	0.3350	0.8377	0.8042
150	0.9563	0.1086	0.9023	0.3482	0.9237	0.9290

### ISIC 2017 dataset

**Fig. 7:**
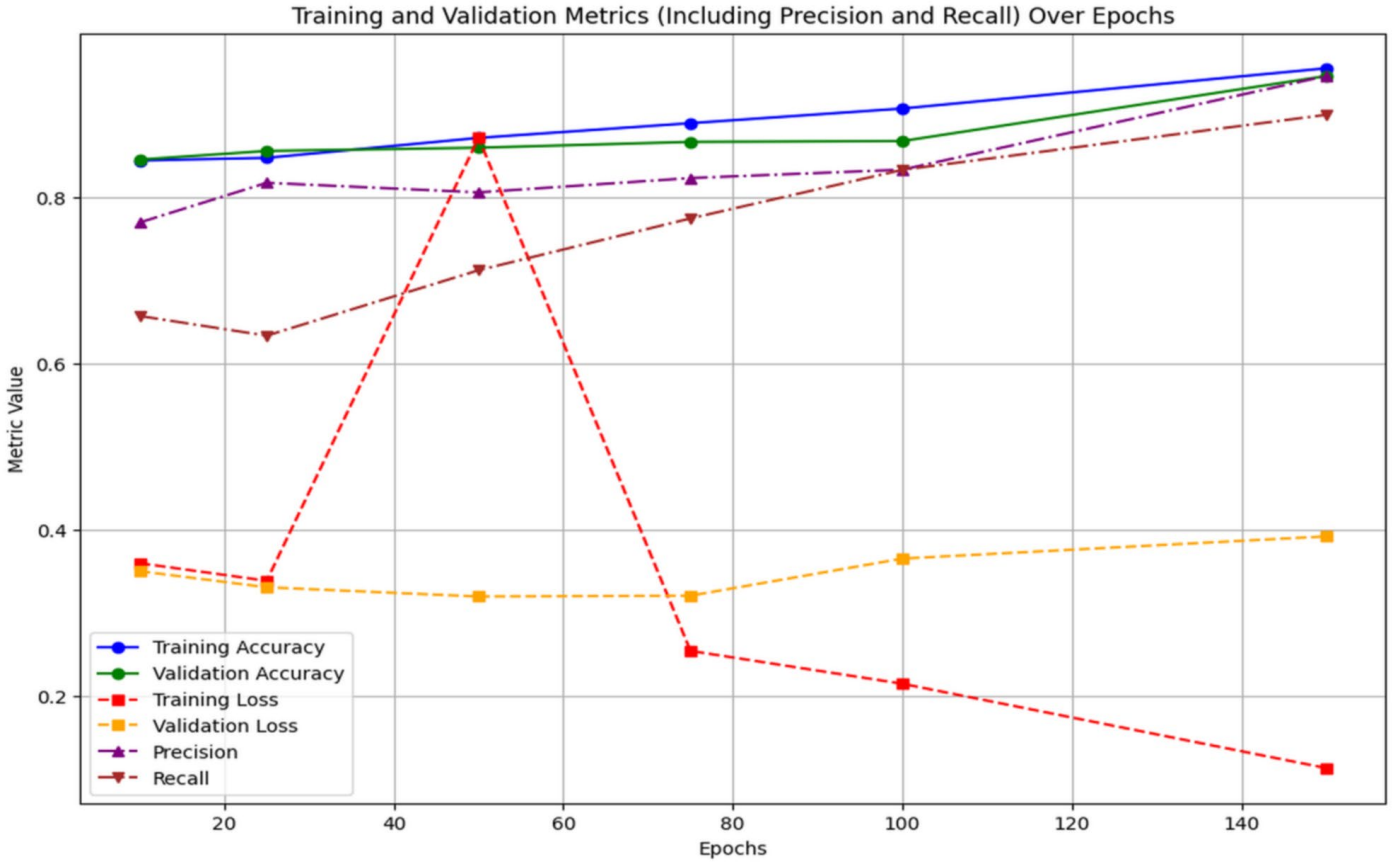
Results of ISIC 2017 Dataset training and validation metrics over 150 epochs.

As training progresses, the model consistently improves across measures to the ISIC-2017 Dataset. Training loss dramatically drops from 0.3600 to 0.1134, while training accuracy rises between 0.8441 at 10 epochs over 0.9550 at 150 epochs. Although validation loss exhibits significant fluctuation, particularly for higher epochs, suggesting possible overfitting, validation accuracy also increases, achieving 0.9021 by 150 epochs. Significant improvements are also shown in precision and recall, which peak around 0.9457 and 0.8991 at 150 epochs, respectively. This development demonstrates how well the model can segment skin lesions from the ISIC 2017 data set, producing consistent outcomes after more training. Performance trends across epochs for ISIC 2017 dataset are reported in Table 6. The training and validation progress for the ISIC 2017 dataset is shown in Fig. 7

**Table 6:** Training and validation performance across epochs on ISIC 2017 Dataset.

Epochs	Training ACC	Training loss	Val—ACC	Val—loss	Precision’s	Recall’s
10	0.8441	0.3600	0.8452	0.3504	0.7695	0.6572
25	0.8473	0.3388	0.8556	0.3310	0.8173	0.6335
50	0.8713	0.8713	0.8595	0.3199	0.8058	0.7122
75	0.8890	0.2545	0.8665	0.3209	0.8230	0.7745
100	0.9066	0.2150	0.8675	0.3655	0.8331	0.8329
150	0.9550	0.1134	0.9021	0.3922	0.9457	0.8991

**Fig. 8:**
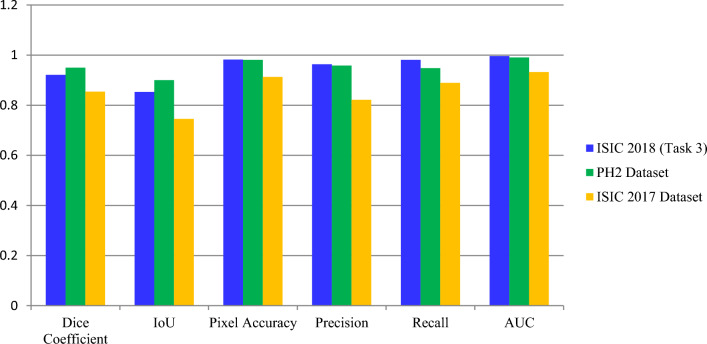
Comparison of metric values across datasets (ISIC 2018, PH2, ISIC 2017).

Cross-dataset validation outcomes for models trained on ISIC 2018 and tested on ISIC 2017 are summarized in Table 7. The segmentation algorithms’ ability to accurately differentiate skin lesions is supported by the quantitative findings obtained from the ISIC 2018, PH2, & ISIC 2017 Datasets. The models’ physically powerful presentation is demonstrated by the metrics obtained, which include the DiceCo-efficient, the Intersection over Union (an IoU), Pixel precision, recall, precision, accuracy, and AUC. The PH2 Dataset, which obtained the finest DiceCo-efficient (0.9500) along with IoU (0.9000), suggests superior segmentation functionality and detail. These findings show the segmentation methods’ generalizability across a variety of Datasets in addition to their resilience. With its potential for clinical use in dermatological diagnostics, wherein accurate and trustworthy segmentation is essential, this flexibility represents a significant advancement in automated skin lesion analysis. Comprehensive quantitative performance of AttenUNeT X across all three benchmark datasets is presented in Table 8. A comparative visualization of segmentation metrics across ISIC 2018, PH2, and ISIC 2017 datasets is given in Fig. 8

**Table 7:** Cross-dataset validation results: Model trained on ISIC 2018 and tested on ISIC 2017.

Metric	ISIC 2018 (Task 3)	ISIC 2017 dataset
Accuracy	0.9824	0.9125
Precision	0.9633	0.8216
Recall	0.9808	0.8894
AUC	0.9961	0.9325
IoU	0.8533	0.7454
DiceCo-efficient	0.9720	0.8542
Loss	0.0429	0.2016

**Table 8:** Quantitative segmentation performance across datasets.

Metric	ISIC 2018 (Task 3)	PH2 dataset	ISIC 2017 dataset
DiceCo-efficient	0.9211	0.9500	0.8542
Intersection over Union (IoU)	0.8533	0.9000	0.7454
Pixel accuracy	0.9824	0.9808	0.9125
Precision	0.9633	0.9583	0.8216
Recall	0.9808	0.9478	0.8894
AUC	0.9961	0.9906	0.9325

**Fig. 9:**
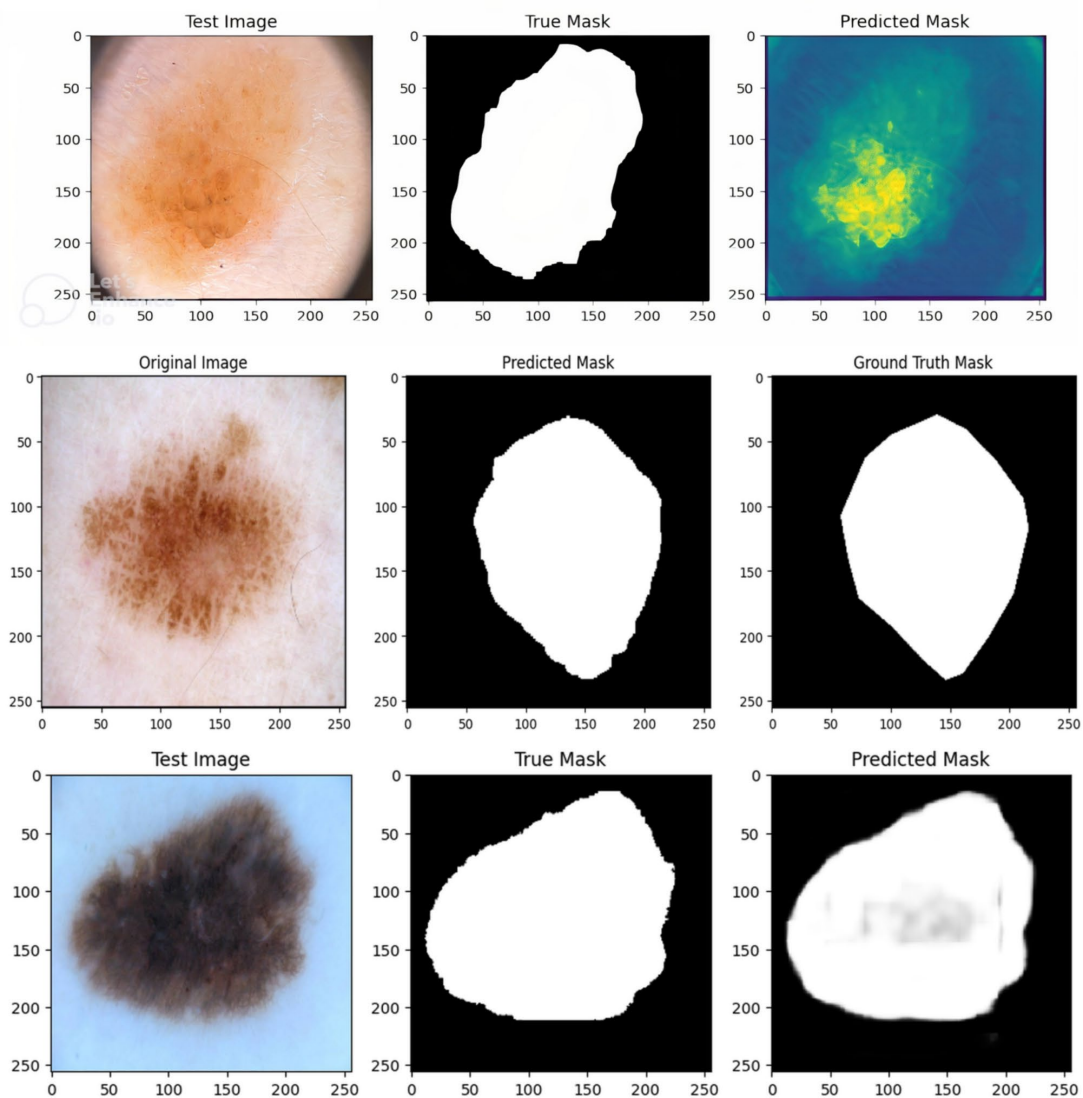
Qualitative segmentation results of AttenUNeT X across ISIC 2017, PH2, and ISIC 2018 Datasets. Error heatmaps highlighting regions of under- and over-segmentation produced by AttenUNeT X on sample test images.

*High DiceCo-efficient****:*** In the ISIC-2018, Dataset, the model’s DiceCo-efficient was 0.9211; for the PH2 Dataset, it was 0.9500; and on the ISIC 2017, Dataset, it was 0.8542. The model well captures the intricate borders of skin lesions, as evidenced by these results, which show significant overlap among the predicted with ground truth lesion masks. *Strong IoU Performance****:*** Exact separation of lesion from non-lesion areas is demonstrated by the model’s IoU values for 0.8533 (ISIC 2018), 0.9000, for instance (PH2), as well as 0.7454 (ISIC 2017). Substantial IoU values throughout Datasets suggest that recall and accuracy are well-balanced. *Robust Pixel Accuracy****:*** The model’s pixel accuracy ratings of 0.9824 for ISIC 2018 alone, 0.9808 on PH2, while 0.9125 for ISIC 2017 demonstrate its high ability to reliably categorize individual pixels. Given its high degree of accuracy, the model appears to be dependable for real-world skin lesion segmentation purpose. Exceptional AUC: Further confirming the model’s possibility of clinical use in dermatology are the AUC metrics of 0.9961 (ISIC 2018, among others), 0.9906 (PH2), & 0.9325 (ISIC 2017), which highlight the model’s efficacy in differentiating among lesion and background classes. Some predicted outputs are displayed as binary lesion masks, while others appear as probability heatmaps or colored overlays. These variations arise from differences in Dataset-specific coding pipelines used to extract results. We retained these representations to demonstrate model performance across multiple visualization styles. Importantly, the underlying segmentation accuracy is evaluated quantitatively (Dice, IoU, Precision, Recall), independent of the visualization style. Hence, heatmaps and binary masks equally confirm the model’s ability to capture lesion boundaries. Qualitative segmentation outputs of AttenUNeT X on representative samples from all datasets are illustrated in Fig. 9

**Fig. 10:**
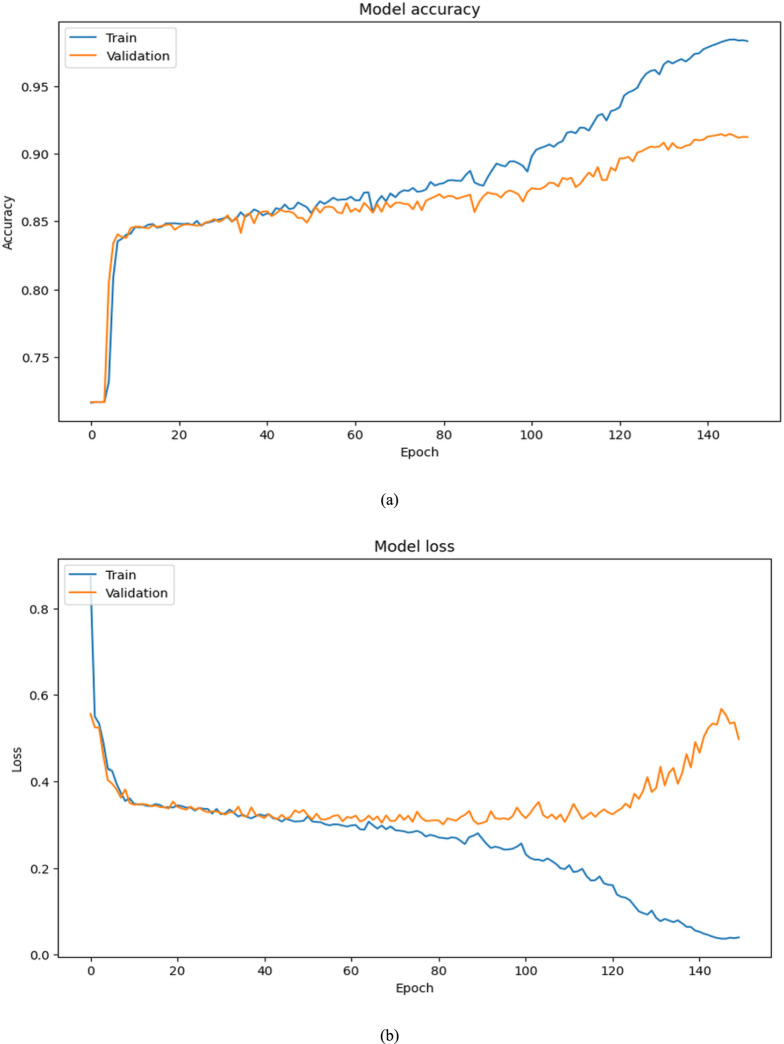
Result of AttenUNeT X model (**a**) Accuracy and (**b**) Loss of ISIC 2018 Dataset.

Each row shows the original image, ground truth mask, and predicted mask. Predicted outputs are displayed in different visualization styles (probability heatmaps, binary masks, colored overlays), depending on the Dataset pipeline. These differences are only visualization choices and do not affect segmentation quality. All evaluations are based on quantitative metrics and boundary accuracy. Figure 10 depicts the accuracy and loss variation of the AttenUNeT X model during ISIC 2018 training..

### Comparison on ISIC-2018 dataset

Our model, AttenUNeT X, demonstrated strong performance on the ISIC 2018 Dataset, setting a high benchmark in skin lesion segmentation. With DiceCo-efficient of 0.9211, IoU of 0.8533, Pixel Acc of 0.9824, Precision 0.9633, Recall 0.9808, and an AUC of 0.9961, AttenUNeT X outperforms or competes closely with other advanced models: From the comparative analysis in Table 9, it is evident that our model, AttenUNeT X, consistently delivers high performance across all evaluation metrics. With a Dice coefficient of 0.9211 and IoU of 0.8533, it outperforms models such as Ms RED (0.8960, 0.8290), FAT-Net (0.9050, 0.8450), GAN-based Segmentation (0.8950, 0.8510), and Boundary-Aware UNet (0.9000, 0.8500). In addition, AttenUNeT X achieves the highest AUC (0.9961), surpassing all compared methods, including FAT-Net (0.9800), Complementary Network (0.9750), and DPFCN (0.9700). Its Pixel Accuracy (0.9824) is also superior to many recent approaches such as MGAN (0.9450) and SSR-UNet (0.9534). While a few models, such as ARCUNet (2025) and MRP-UNet (2024), report competitive Dice values, their overall performance across multiple metrics is either incomplete or slightly below our framework. A detailed quantitative comparison of AttenUNeT X with state-of-the-art segmentation models on the ISIC 2018 dataset is provided in Table 9.

**Table 9:** Comparison of AttenUNeT X with state-of-the-art segmentation methods on ISIC 2018 Dataset.

Model	Dice coefficient	IoU	Pixel accuracy	Precision	Recall	AUC
AttenUNeT X	0.9211	0.8533	0.9824	0.9633	0.9808	0.9900
ARCUNet^19^	0.9688	NA	0.9819	NA	NA	0.9353
SSR-UNet^20^	0.9021	0.7138	0.9534	0.9754	0.8849	NA
MRP-UNet^30^	0.9236	0.9128	0.9551	0.9344	0.8857	NA
Ms RED^25^	0.8960	0.8290	0.9602	0.9000	0.9000	N/A
FAT-Net^27^	0.9050	0.8450	0.9600	0.9150	0.9030	0.9800
Improved Tuna Swarm (2023)	0.8725	0.8350	0.9120	0.8940	0.8810	N/A
MGAN^18^	0.9010	0.8360	0.9450	0.9550	0.9360	NA
GAN-based segmentation (2023)	0.8950	0.8510	0.9230	0.9065	0.8920	0.9400
Complementary network^26^	0.9120	0.8500	N/A	0.9200	0.9100	0.9750
Dual-attention U-Net^13^	0.8900	0.8200	N/A	0.8800	0.8750	0.9600
DPFCN^34^	0.9100	0.8300	0.9500	0.9010	0.8900	0.9700
Boundary-aware method^11^	0.9000	0.8500	0.9450	0.8970	0.8850	0.9600
SK-Net^35^	0.8800	0.8100	0.9400	0.8800	0.8700	0.9500
UNeT + +^33^	0.8900	0.8300	0.9480	0.8900	0.8800	0.9500

**Fig. 11:**
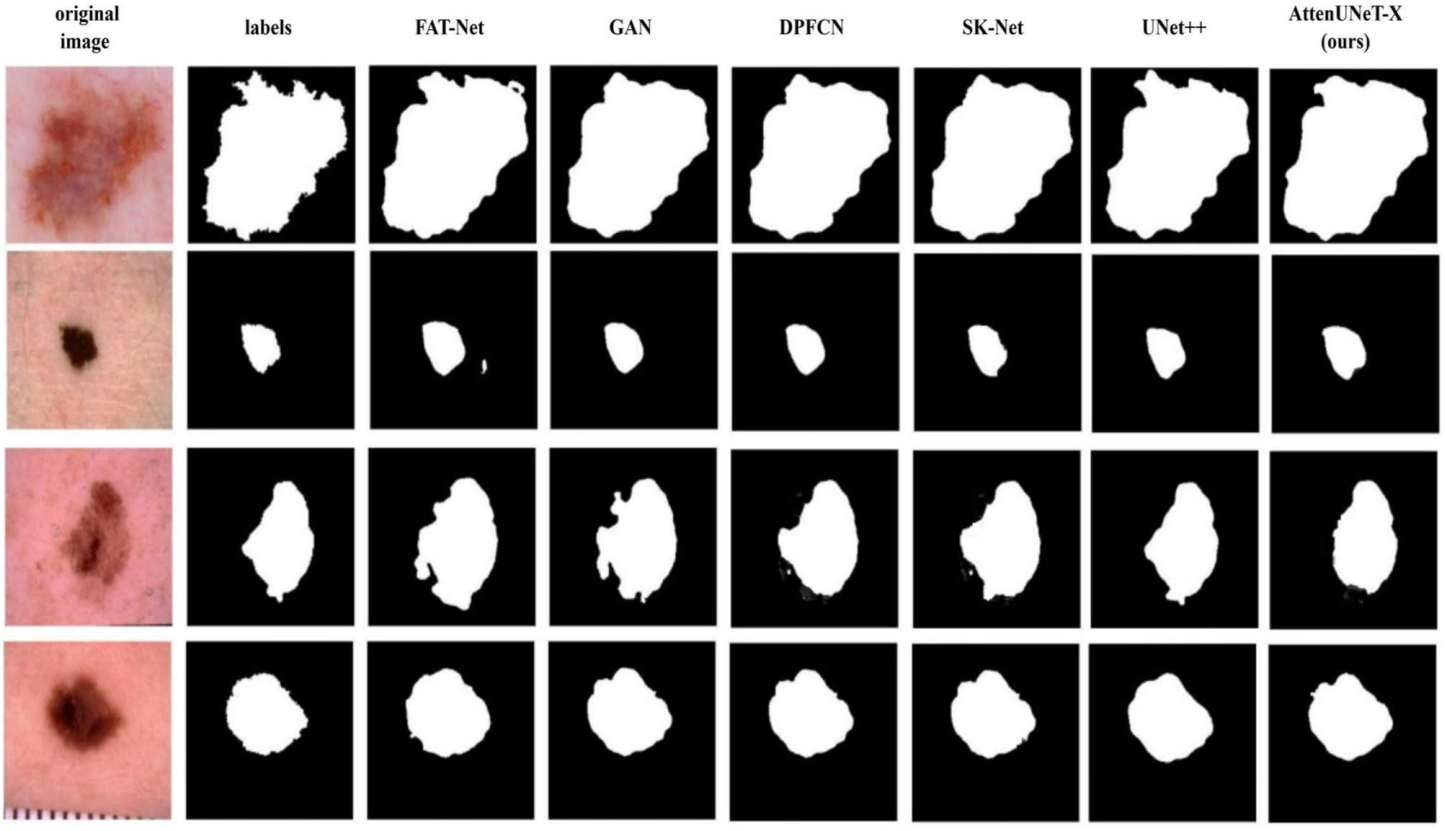
Visual overlap comparison of segmentation outputs. AttenUNeT X predictions align closely with ground truth boundaries, outperforming competing models in preserving fine lesion details. Summary visualization showing model performance across datasets and potential areas for improvement.

This highlights that AttenUNeT X not only maintains strong balance across all measures but also sets a high benchmark in segmentation accuracy, boundary localization, and lesion detection reliability. These comparisons highlight that AttenUNeT X not only excels in segmentation accuracy but also provides robust boundary detection and lesion localization. The high scores across all metrics reinforce its potential for clinical applications in dermatology. Visual overlap comparisons between predicted masks and ground truths highlighting lesion boundary alignment are illustrated in Fig. 11.

### Comparison on PH2 dataset

Our AttenUNeT X model showed remarkable performance on the PH2 Dataset, achieving a DiceCo-efficient of 0.9500, IoU of 0.9000, Pixel Accuracy 0.9808, Precision 0.9583, Recall 0.9478, and an AUC of 0.9906. Compared to other advanced models, AttenUNeT X maintains a competitive edge: The results in Table 10 demonstrate that AttenUNeT X achieves superior performance across almost all evaluation metrics. With a Dice coefficient of 0.9500 and IoU of 0.9000, our model surpasses FAT-Net (0.9100, 0.8600), GAN-based Segmentation (0.8950, 0.8580), Boundary-Aware Method (0.9200, 0.8700), and UNeT + + (0.9100, 0.8600). Although MRP-UNet (2024) reports a comparable Dice (0.9419) and IoU (0.9077), it falls slightly short of AttenUNeT X’s overall consistency. In terms of Pixel Accuracy, AttenUNeT X records 0.9800, higher than Boundary-Aware UNet (0.9500), Dual-Attention U-Net (0.9480), and UNeT + + (0.9520). The model also secures one of the strongest Precision scores (0.9580), outperforming methods such as FAT-Net (0.9050) and Boundary-Aware UNet (0.9080), while its Recall of 0.9470 strikes an optimal balance compared to SSR-UNet (0.9754), which favors recall but compromises IoU and overall stability. Based on the PH2 Dataset, these findings position AttenUNeT X as a top model in skin lesion segmentation. The model’s resilience and dependability for dermatological diagnoses are demonstrated by its outstanding AUC, high Dice with IoU scores, and exceptional boundary correctness. Table 10 presents a comprehensive comparison of AttenUNeT X with other recent architectures evaluated on the PH2 dataset.

**Table 10:** Comparison of AttenUNeT X with state-of-the-art segmentation methods on PH2 Dataset.

Model	Dice coefficient	IoU	Pixel accuracy	Precision	Recall	AUC
AttenUNeT X	0.9500	0.9000	0.9800	0.9580	0.9470	0.9900
SSR-UNet^20^	0.9021	0.7138	0.9530	0.9754	0.8849	NA
UCM-Net^31^	0.9015	0.8762	0.8883	0.9064	0.8791	NA
FDUM-Net^32^	0.8981	0.8845	0.9053	0.9173	0.8811	NA
MRP-UNet^30^	0.9419	0.9077	0.9613	0.9265	0.9192	NA
FAT-Net^27^	0.9100	0.8600	0.9550	0.9050	0.8870	0.9700
GAN-based Segmentation (2023)	0.8950	0.8580	0.9230	0.9060	0.8920	0.9400
Improved Tuna Swarm U-EfficientNet^28^	0.9300	0.8800	N/A	0.9150	0.9000	0.9700
Boundary-aware method^11^	0.9200	0.8700	0.9500	0.9080	0.8900	0.9700
Dual-attention U-Net^13^	0.9000	0.8500	0.9480	0.8900	0.8800	0.9600
UNeT + +^33^	0.9100	0.8600	0.9520	0.8990	0.8900	0.9600

## Discussion

The proposed AttenUNeT X model demonstrates strong performance in dermoscopic skin lesion segmentation by integrating decoder feedback refinement, statistical feature enhancement, and attention-based localization. Unlike conventional architectures such as U-Net or DeepLabV3 + , the inclusion of an Order Statistics Layer and decoder-level feedback enables the recovery of fine lesion boundaries and subtle features often missed by baseline approaches. Our evaluation across ISIC 2018, ISIC 2017, and PH2 confirms both high segmentation accuracy and cross-dataset generalizability. Despite the limited sample size of PH2, the model maintains robust performance, suggesting resilience to domain shift. Nevertheless, we acknowledge the risk of overfitting on small datasets and plan to address this by incorporating additional sources such as Derm7pt Clinical and Dermoscopic Skin Lesion Dataset (Derm7pt), Human Against Machine 10,000 Dataset (HAM10000), and Skin Disease 198 (SD-198) in future work. Clinical relevance represents another strength of AttenUNeT X. Accurate lesion boundaries are critical for dermatologists in assessing asymmetry, border irregularity, and pigment distribution, which are key diagnostic indicators for melanoma. By producing precise and interpretable masks, the model could be integrated into clinical decision-support systems or dermoscopic image viewers to assist with real-time triage or second-opinion validation. Furthermore, the modular design of AttenUNeT X, built on a lightweight U-Net backbone with selective enhancements, facilitates deployment in resource-constrained environments such as mobile platforms or portable diagnostic devices. Although we did not directly re-implement DeepLabV3 + or DenseNet-UNet due to resource constraints, community benchmarks indicate that DeepLabV3 + with a ResNet-50 backbone requires ~ 26–42 M parameters, while DenseNet-UNet variants remain parameter-heavy owing to dense connectivity. In contrast, AttenUNeT X achieves competitive segmentation accuracy with a notably smaller memory footprint, making it more suitable for deployment in real-world clinical environments.

## Conclusion 

In this paper, we proposed AttenUNeT X, an enhanced deep learning architecture for accurate skin lesion segmentation. By incorporating decoder feedback, a custom Order Statistics Layer, and attention modules into the U-Net framework, the model addresses key challenges in lesion boundary extraction and contextual misclassification. Experimental validation on ISIC 2018, ISIC 2017, and PH2 confirms the effectiveness of AttenUNeT X, achieving superior Dice scores and visual accuracy compared to several methods. Ablation studies further support the contribution of each proposed component. For future work, we aim to extend the model toward multi-task learning, enabling simultaneous lesion segmentation and classification. Incorporating transformer blocks will be explored to capture long-range dependencies, and cross-domain evaluations will be conducted on datasets with rare lesion types and diverse skin tones. Optimization for real-time clinical deployment via web-based platforms and mobile diagnostic tools will also be prioritized. In addition, while standard augmentation methods such as rotation, flipping, scaling improve dataset diversity, they remain limited in modeling rare pathological variations. Generative adversarial networks (GANs) and diffusion-based approaches may be employed to synthesize realistic lesions, further enhancing robustness. Given recent advances in hybrid CNN–Transformer architectures like TransUNet, Swin-UNet, extending AttenUNeT X with such modules forms a promising direction for future research. Overall, AttenUNeT X represents a step toward building robust, interpretable, and deployable deep learning models for dermatological diagnostics.

## Limitations

The datasets used in this study primarily include common lesion types and may lack representation of rare conditions and diverse skin tones, which could limit generalizability. Moreover, curated benchmark datasets may not fully capture real-world variability, such as inconsistent lighting, imaging artifacts, and heterogeneous acquisition devices. While AttenUNeT X demonstrates good cross-dataset performance across ISIC 2017, ISIC 2018, and PH2, domain shifts remain a challenge, particularly when applied to under-represented populations or rare conditions. We also note that some recent methods, such as ARCUNet (0.9688 Dice) and MRP-UNet (0.9236 Dice), report slightly higher DSC values. However, AttenUNeT X achieves a stronger overall balance across Dice, IoU, Precision, Recall, and AUC. Our design emphasizes lesion boundary refinement and interpretability rather than maximizing a single metric. Finally, although the proposed architecture reduces computational complexity relative to other advanced networks, additional strategies such as ensemble learning and post-processing may further improve performance. Future research will explore these directions to enhance both accuracy and robustness.

## Data Availability

The Datasets used in this research are openly accessible and include: ISIC Datasets (ISIC 2018 and ISIC 2017) **:** Available at the [ISIC Archive] (https://www.isic-archive.com/), PH2 Dataset **s** Provided as a public Dataset in the dermatology

## Abbreviations

ISIC: International skin imaging collaboration
DL: Deep learning
CNN: Convolutional neural network
ROI: Region of interest
PH2: Pedro Hispano hospital dataset
